# Hip-sacroiliac joint-spine syndrome in total hip arthroplasty patients

**DOI:** 10.1038/s41598-024-54472-4

**Published:** 2024-02-15

**Authors:** Ayumi Kaneuji, Makoto Fukui, Eiji Takahashi, Yusuke Sanji, Hiroaki Hirata, Norio Kawahara

**Affiliations:** Department of Orthopaedic Surgery, Kanazawa Medical University, 1-1 Daigaku, Uchinada, Kahoku-gun, Ishikawa 920-0293 Japan

**Keywords:** Diseases, Medical research

## Abstract

This study is designed to compare the extent of sacroiliac joint (SIJ) degeneration at total hip arthroplasty (THA) for two pathologies: osteoarthritis of the hip (OA) and osteonecrosis of the femoral head (ON). We also assessed the prevalence of SIJ degeneration in patients with lumbar spondylolisthesis or degenerative scoliosis. A total of 138 hips from 138 patients (69 OA and 69 ON) were assessed in this study, including 66 hips affected by OA secondary to developmental dysplasia of the hip. The degenerative changes in the SIJ and lumbar spine were evaluated prior to THA using radiographs and computed tomography (CT) scans, showing 9 instances of spondylolisthesis and 38 of degenerative scoliosis. The OA group exhibited longer duration from onset to surgery than the ON group. The OA group also included more cases with significant pelvic obliquity (3 degrees or more) and with significant increases in SIJ sclerosis and irregularities. Patients with lumbar spondylolisthesis or degenerative scoliosis were significantly more likely to have SIJ irregularities. The prevalence of SIJ degeneration was higher in cases of THA for OA than for ON. This study also suggests the possibility of Hip-SIJ-Spine syndrome in THA patients with OA.

## Introduction

Hip-spine syndrome was first described by Offierski and Macnab in 1983^1^. It is classified into four types: simple, complex, mixed, and misdiagnosed. The simple type involves degenerative changes in either the hip joint or the spine, with that degeneration being the primary cause of the condition. The complex type involves degenerative changes in both the hip joint and the spine, with both contributing to the pathology. The mixed type refers to the interaction between hip and spinal pathologies, so that the conditions of the hip joint and the spine mutually affect each other. The misdiagnosed type occurs when incorrect treatment is provided due to a lack of awareness of the involvement of both hip and spine pathologies. An anatomical study also has detailed the mutual influence of degenerative changes in the spine and the hip joint^2^.

In recent years, researchers have suggested that lumbosacral fusion and long fusion of the lumbar spine can lead to progressive hip osteoarthritis (hip OA)^3,4^. Additionally, sacroiliac joint (SIJ) fixation has been linked to a two-fold increase in the risk of dislocation after total hip arthroplasty^5^, suggesting a mechanical influence on the SIJ between the lumbar spine and the hip joint. Credible reports indicate that 14–30% of low back pain originates from the SIJ^7–9^, and extensive study of the nerve supply to the SIJ suggests that not only the SIJ capsule but also the surrounding tissues such as ligaments can manifest pain^10–18^. However, the origin of SIJ pain remains unclear^6,16,17^. While excessive motion is restricted by ligaments, the SIJ joint has some limited mobility^6^, and increased instability or stress on the posterior ligaments of the SIJ may cause pain and dysfunction. In fact, symptomatic relief has been reported from immobilization by external fixation of the pelvis or injections into the posterior ligaments^19,20^.

There have been reports of increased stress on the SIJ due to lumbosacral fusion^21,22^, as well as reports of increased degeneration of the SIJ^23^, strongly indicating an interrelationship between the lumbar spine and the SIJ, and a link between hip OA and SIJ dysfunction has been suggested^24,25^. Asada et al.^25^ analyzed preoperative CT scans of patients with hip OA who underwent total hip arthroplasty (THA) and noted a significantly higher incidence of narrowed joint space, osteophyte formation, and vacuum phenomena in the SIJ compared with a control group. Elucidating the relationship between the hip, spine, and SIJ disorders is thus of considerable clinical importance.

In this study, we formulated two hypotheses: first, that long-term degeneration of the hip joint has a major impact on the SIJ, making it more susceptible to Hip-SIJ syndrome, and second, that patients with the condition termed “Hip-Spine syndrome” show a higher prevalence of SIJ degeneration, suggesting the existence of what we have designated as “Hip-SIJ-Spine syndrome”. We then conducted a comparative study of patients undergoing THA for hip OA and for idiopathic osteonecrosis of the femoral head (ON).

THA for OA is often performed after a long duration of illness, while ON frequently requires THA within a short timeframe. This is the reason for selecting these two conditions for comparison. Furthermore, in our facility, a significant proportion of OA cases are associated with developmental dysplasia of the hip (DDH), and ON often occurs in the unaffected hip joint, making it easier to compare and study in the context of this research.

## Methods

The subjects of this study were patients who underwent THA for the treatment of OA or ON. A total of 93 patients (100 hips) who received THA for OA between June 2016 and December 2016 were enrolled. Since there was a possibility of changes in findings after unilateral surgery, seven cases of bilateral procedures were included only for analysis of the first operated side. An additional 24 patients (24 hips) were excluded because of inadequate CT confirmation of SIJ, so that 69 patients (69 hips) were included in analysis for the OA group. Because the annual number of cases of ON is relatively small, the treatment period for ON was extended from January 2010 to October 2022, and 83 patients (109 hips) were enrolled. Of those, 26 bilateral cases were counted on one side only. Seven patients (7 hips) were excluded because the patient had already undergone THA on one side, and an additional seven patients (7 hips) were excluded because of inadequate confirmation of the SIJ by CT or because of significant collapse of the necrotic area from advanced OA. This resulted in 69 cases (69 hips) that were included in analysis for the ON group.

Patient demographics are shown in Table 1. There were no significant differences in age, height, weight and BMI between the groups. However, due to a higher prevalence of OA resulting from developmental dysplasia of the hip (DDH), female patients made up a significantly higher portion of the OA group (87%) than of the ON group (55%). In the OA group, only 3 hips were classified as primary OA, while 66 hips (96%) were classified as secondary OA from DDH. Crowe classification Group I accounted for 61 hips (92%), indicating a relatively low degree of hip dislocation in this group. In the ON group, 38 hips (55%) were classified as Group 4, indicating narrowing of the joint space but not reaching terminal OA.

**Table 1:** Demographic data for all patients.

	OA	ON	*p* value
Cases	69	69	NS
Hips	69	69	NS
Female : Male	60 : 9	38 : 31	< 0.01
Right : Left	38 : 31	39 : 30	NS
Age (years)*	62.0 (44–87)	59.4 (33–81)	NS
Height (cm)*	156.3 (142.6–181.0)	160.2 (140.0–178.6)	NS
Weight (kg)*	58.5 (36.8–98.8)	61.5 (40.8–99.0)	NS
BMI (kg/m^2^)*	23.9 (15.6–40.7)	23.9 (14.6–36.8)	NS
Tönnis grade (0:1:2:3)	0:0:7:62	NA	
Crowe classification^34^ (group I:II:III:IV)	61:5:0:0	NA	
Stage classification^35^ for ON (1:2:3:4)	NA	0:6:25:38	

*OA* osteoarthritis of the hip joint, *ON* osteonecrosis of the femoral head, *BMI* body mass index.

*Values are mean and range, *NA* not applicable, *NS* not significant.

Preoperative leg length discrepancy was measured by assessing the difference in Spina Malleolar Distance between the left and right sides before surgery, and was classified as an absolute value of ≤ 5 mm or ≥ 6 mm. A difference of ≥ 6 mm was defined as leg length discrepancy.

### Radiographic and CT analysis

The following items were investigated using imaging:

I. Preoperative anteroposterior pelvic radiographs

1. Pelvic tilt angle: Confirmation of the presence of lateral tilt of the iliac wings, with a tilt of 3 degrees or more in either direction classified as tilt present.
2. Discrepancy in the obturator foramen: Confirmation of any obvious left–right asymmetry in the shape of the obturator foramen.

II. Preoperative frontal and lateral lumbar spine images

1. Lumbar scoliosis: Confirmation of the presence of lumbar scoliosis and measurement of the Cobb angle. A Cobb angle of 5 degrees or more was classified as scoliosis present.
2. Lumbar degenerative spondylolisthesis: Presence of more than 25% slip of a lumbar vertebra was classified as spondylolisthesis present.

III. Preoperative CT images

SIJ degenerative changes^26,27^: CT cross-sectional images with a slice interval of 1 mm were examined for the following:

1. Sclerosis: Presence of sclerosis adjacent to either the sacral or iliac side of the SIJ was classified as sclerosis present.
2. Vacuum phenomenon: Presence of CT attenuation similar to air in the joint space of the SIJ was classified as vacuum phenomenon present.
3. Discrepancy in joint space width: Confirmation of asymmetry in the width of the SIJ space was classified as discrepancy in joint space width.
4. Irregularities: Slight narrowing of the SIJ space, non-parallel joint surfaces of the sacral or iliac side, or erosive changes in a portion of the joint surface were classified as irregularities present.
5. Discrepancy in iliac wing opening angle: Measurement of the angle formed by drawing a perpendicular line from the midline sacral ridge to the vertebral body at the second sacrum level and drawing a line from the midpoint of the width of the most anterior part of the iliac wing visible on the CT slice to the sacral ridge. A difference in absolute values of 3 degrees or more between the left and right sides was classified as iliac wing opening angle discrepancy present.

### Statistical analysis

Statistical analysis was performed using the chi-square test or Fisher's exact test by Microsoft Excel. A p-value of 0.05 or less was considered statistically significant.

All methods in this study were performed in accordance with the relevant guidelines and regulations, and approval was granted by the Ethics Committee of Kanazawa Medical University.

### Ethical approval

Approval was granted by the Ethics Committee of Kanazawa Medical University.

### Consent to participate

Written informed consent was obtained from the all patients.

## Results

The OA group differed significantly from the ON group in several parameters. Significantly more prevalent in the OA group were duration of illness, angle of pelvic outward tilt, instances of pelvic outward tilt of 3 degrees or more, and sclerotic and irregular images in the SIJ (Table 2). No significant intergroup differences were noted in the frequency of lumbar spondylolisthesis and scoliosis. However, the presence of lumbar spondylolisthesis and of 5 degrees or more of scoliosis were significantly associated with a higher prevalence of irregular SIJ images (*P* = 0.027) and (*P* = 0.00373), respectively (Table 3). Further analysis revealed that the presence of SIJ irregularities was significantly associated with asymmetry in the SIJ joint space in the entire group (*P* < 0.0001). No significant differences were observed in other parameters.

**Table 2:** Comparison of OA and ON groups.

	OA	ON	*p* value
Duration from onset to surgery (years)^#^	5.0 (0.25–20)	0.75 (0.1–24)	< 0.01
Leg length discrepancy (LLD) at surgery (mm)*	10.0 (0–40)	5.7 (0–20)	0.409
LLD > = 6 mm (yes:no)	44:25	26:43	0.07
Lateral inclination angle of pelvis (°)*	1.6 (0–9)	0.7 (0–11)	< 0.01
Lateral inclination angle of pelvis > = 3°(yes:no)	25:44	6:63	< 0.01
Asymmetry of obturator foramen (yes : no)	52:17	7:62	0.198
Spondylolisthesis (cases)	8:61	1:68	0.374
Cobb angle at lumbar spine (°)*	3.5 (0–17)	2.9 (0–26)	0.121
Cobb angle > = 5°(yes:no)	20:49	18:51	0.751
Sclerotic change of SIJ (yes:no)	30:39	14:55	< 0.01
Vacuum phenomenon of SIJ (yes:no)	32:37	17:52	0.828
Asymmetry of SIJ space (yes:no)	20:49	7:62	0.464
Irregularity of SIJ (yes:no)	38:31	9:60	< 0.01
Angle of right iliac wing (°)*	44.7 (34–58)	45.8 (36–58)	0.464
Angle of left iliac wing (°)*	44.9 (33–59)	45.5 (34–58)	0.134
Difference in bilateral iliac wing angle (°)*	4.5 (0–25)	3.5 (0–15)	0.598

*SIJ* sacro-iliac joint, *OA* osteoarthritis of the hip joint, *ON* osteonecrosis of the femoral head.

^#^Values are median and range. *Values are mean and range.

Statistical analysis for continuous values was performed using a t-test. A chi-square test was conducted for the other variables.

**Table 3:** Relationship between lumbar spine degeneration and SIJ degeneration.

		Spondylolisthesis			Cobb angle > = 5°		
		Yes (cases)	No (cases)	*p* value	Yes (cases)	No (cases)	*p* value
Irregularity of SIJ	Yes	7	40	0.027	15	32	0.0037
	No	2	89		23	68	
Sclerotic change in SIJ	Yes	5	47	0.35	13	39	0.5733
	No	4	82		25	61	
Asymmetry of SIJ space	Yes	5	22	0.518	7	20	0.0861
	No	4	107		31	80	
Asymmetry of obturator foramen	Yes	3	21	0.482	7	17	0.2877
	No	6	108		31	83	
Vacuum phenomenon in SIJ	Yes	5	45	0.485	12	38	0.6745
	No	4	84		26	62	
Lateral inclination of pelvis > = 3°	Yes	5	78	0.122	22	61	0.2769
	No	4	51		16	39	

*SIJ* sacro-iliac joint, Fisher's exact test was conducted for all analyses.

**Figure 1:**
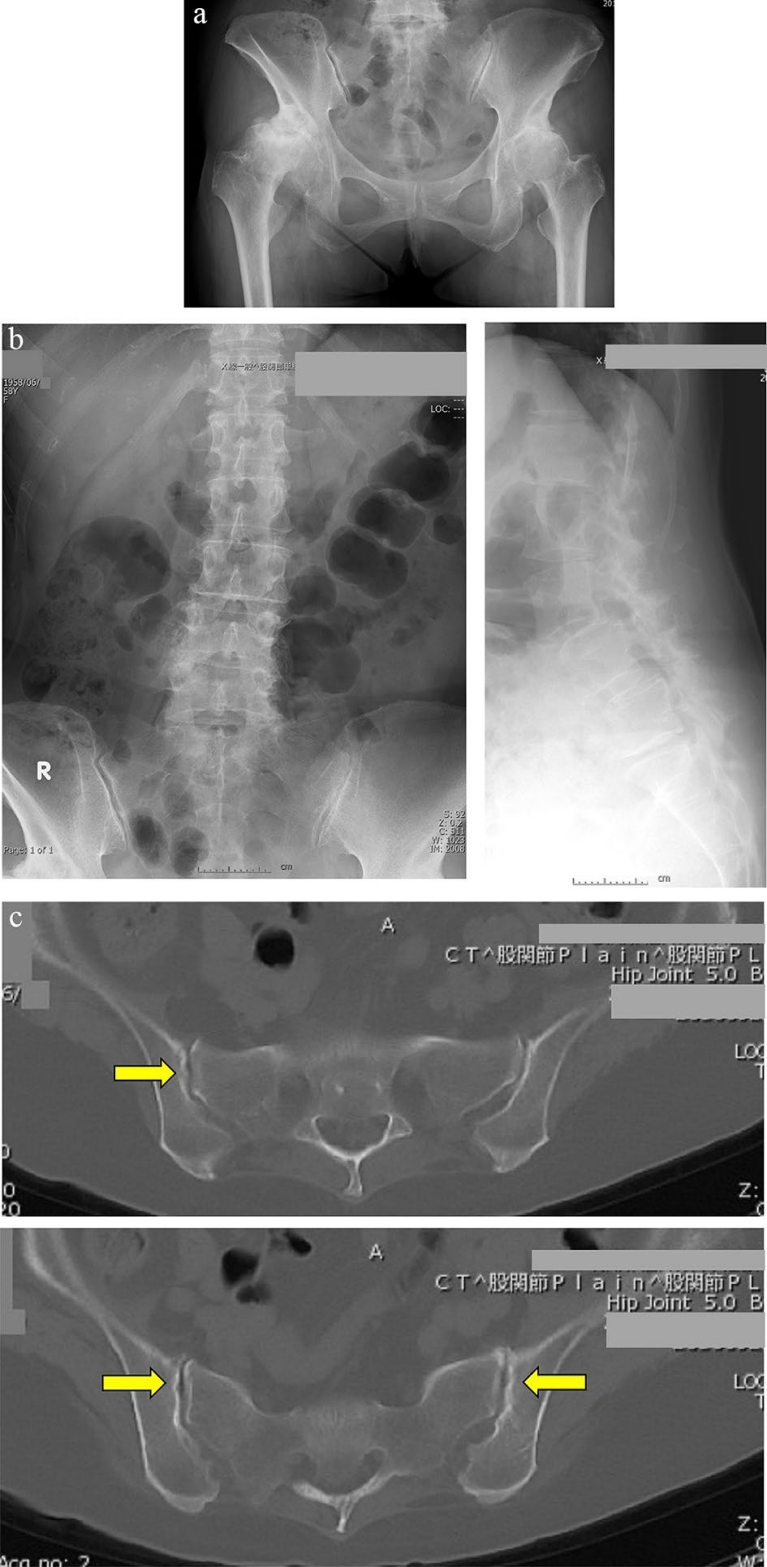
Hip OA secondary to DDH. (**a**) Pre-operative x-ray. The patient had bilateral terminal-stage OA of the hip joint. The SIJ space differed visibly between right and left sides. (**b**) Prior to left-side surgery, the patient was diagnosed with a 25% slip of the fourth lumbar vertebra indicative of spondylolisthesis. Scoliosis with a Cobb angle of 7 degrees was also observed. (**c**) CT imaging showed SIJ irregularities and the presence of vacuum phenomena (yellow arrows). OA: osteoarthritis, DDH: developmental dysplasia of the hip, SIJ: sacroiliac joint, CT: computed tomography.

*Case study 1* (Fig. 1a–c): A 58-year-old female presented with bilateral hip OA. She had been experiencing bilateral hip pain for the past 10 years. The pain gradually worsened, leading to limited range of motion, and she underwent left THA. Subsequently, a right THA was also performed.

**Figure 2:**
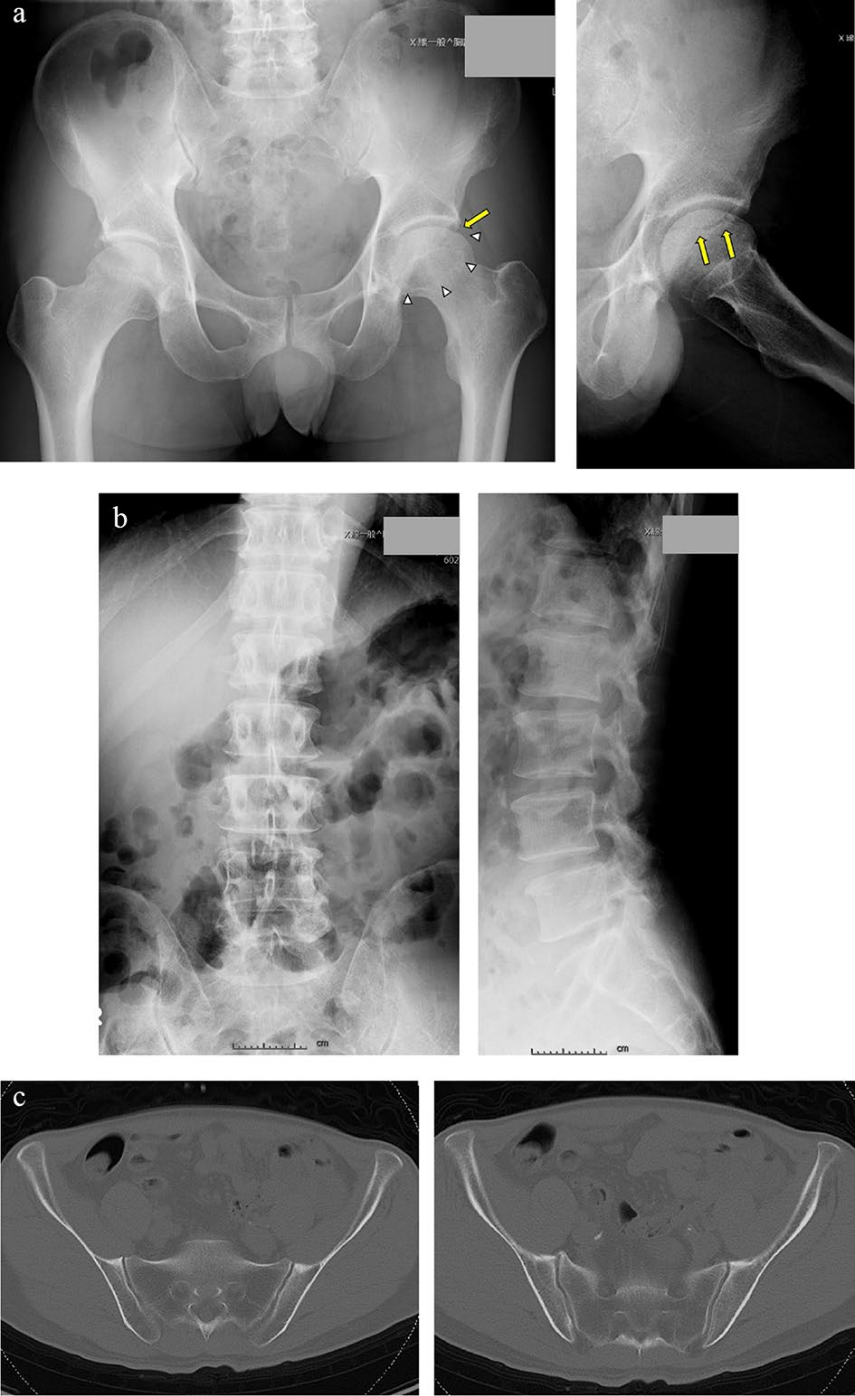
Alcohol-induced ON. (**a**) The sclerotic boundary was visible (white triangles), with bone collapse on the left femoral head (yellow arrows). (**b**) X-ray images showed no lumbar spondylolisthesis except for slight scoliosis. (**c**) CT images showed no abnormalities, including SIJ irregularities. ON: osteonecrosis of the femoral head, SIJ: sacroiliac joint, CT: computed tomography.

*Case study 2* (Fig. 2a–c): A 51-year-old male presented with left hip pain continuing for the past 3 months and was diagnosed with stage 3 ON on the left side. Due to severe pain, he underwent left THA.

## Discussion

The results of this study suggested that THA was associated with significantly more degenerative change in the SIJ of the OA group than the ON group. In addition, the presence of irregularities in the SIJ was significantly associated with greater asymmetry in the SIJ space, indicating that excessive load on the SIJ due to hip OA may have led to degenerative changes^24,25^. This study showed little correlation with leg length discrepancy, suggesting that SIJ degeneration may progress even in the absence of conditions such as DDH-related leg shortening. However, it is also possible that DDH itself increases stress on the SIJ. Toyohara et al. analyzed SIJ stress using finite element models based on CT images before and after periacetabular osteotomy in four DDH patients^28^. They reported that stress on the SIJ and the posterior sacroiliac ligament, which is commonly observed in preoperative dysplastic hips, often decreased after surgery, indicating that excessive load on the SIJ was reduced after formation of a near-normal acetabulum. This suggests that the morphology of DDH itself may increase the load on the SIJ.

A significant association was noted between SIJ joint irregularities and the presence of more than 25% of lumbar spondylolisthesis or scoliosis of 5° or more, indicating a higher prevalence of SIJ degeneration in the patient group that underwent THA and had degenerative spinal diseases. Kwon et al. reported a higher prevalence of SIJ degeneration in patients with spinopelvic imbalance compared to those with lumbar spinal canal stenosis (LSCS)^29^, and Chen has stated that SIJ degeneration was more common in patients with spondylolisthesis^30^. Although a systematic review has shown no consensus regarding SIJ degeneration after lumbar spinal fixation^31^, there has been some suggestion that the SIJ degeneration described in the previous reports^24,25,30^ might have been caused by spinal disease or hip OA. However, our study showed SIJ degeneration in some cases of Hip-Spine syndrome, in which spinal degeneration is associated with hip OA. Although further investigation is needed in a larger number of clinical cases, of course, with careful consideration of clinical symptoms, the new concept of “*Hip-SIJ-Spine syndrome*” should be focused on patients with Hip-Spine syndrome.

This study indicated that long-term degeneration of the hip joint has a major impact on the SIJ, making it more susceptible to Hip-SIJ syndrome. And patients with the condition termed “Hip-Spine syndrome” may show a higher prevalence of SIJ degeneration, suggesting the existence of what we have designated as “*Hip-SIJ-Spine syndrome”*.

This study did not make use of the SIJ degeneration score developed by Backlund et al. or Eno's classification^32,33^. This was because previous reports on the relationship between hip OA and SIJ degeneration^25^ showed no significant difference in scores between the OA and control groups, and data on osteophyte levels were contradictory. In the present study, SIJ joint irregularities, which were not included in the SIJ degeneration score, were found to be associated with hip OA. SIJ joint irregularities are often observed on CT scans as early changes and may be interpreted as the first imaging of SIJ degeneration^27^. Considering that SIJ instability can induce symptoms^19^, these changes may also represent the first imaging of instability. Further investigation is needed in the future.

This study had several limitations. First, observations were limited to cases that underwent THA, and it is unclear whether similar occurrences of Hip-SIJ-Spine syndrome are more likely in cases of DDH or femoroacetabular impingement. Second, no data is currently available on whether SIJ degeneration is similarly likely to occur in simple Hip-Spine syndrome where pathology extends from the lumbar spine to the hip. Third, clinical symptoms were not confirmed, so it is unclear whether the concept of Hip-SIJ-Spine syndrome is clinically relevant or clearly expressed. Fourth, cases of DDH-related hip OA predominated in this study, and we do not know whether the same pathology would occur in cases of primary hip OA. Fifth, the sample size was small.

Diagnosing SIJ dysfunction was not easy in the past, but it has been facilitated by the SIJ scoring system developed by Kurosawa et al.^18^ and based on physical examination and pain region. The SIJ score has a total of 9 possible points, including 2 points for groin pain. In this context, for physicians working with OA patients who require THA, our findings may be useful clinically as a warning that groin pain should be differentiated from SIJ if the spine also has degenerative disease. Further research is warranted.

## Data Availability

The datasets analyzed during this given study are available from the corresponding author on reasonable request.
